# Poisonous Mushrooms

**Published:** 1856-05

**Authors:** 


POISONOUS MUSHROOMS.—When mushrooms are prepared, an onion is to be put into the vessel and boiled or fried with them. If the onion retain its white color, the mushroom may be eaten without any fear; but if the onion turns blue or black, you may be sure that all or at least part of the mushrooms are poisonous.

